# Changes and net ecosystem productivity of terrestrial ecosystems and their influencing factors in China from 2000 to 2019

**DOI:** 10.3389/fpls.2023.1120064

**Published:** 2023-03-15

**Authors:** Yutao Huang, Fang Wang, Lijuan Zhang, Junfang Zhao, Hong Zheng, Fan Zhang, Nan Wang, Jiakai Gu, Yufeng Zhao, Wenshuai Zhang

**Affiliations:** ^1^ Heilongjiang Province Key Laboratory of Geographical Environment Monitoring and Spatial Information Service in Cold Regions, Harbin Normal University, Harbin, China; ^2^ State Key Laboratory of Severe Weather, Chinese Academy of Meteorological Sciences, Beijing, China; ^3^ Laboratory of Climate Application, Climate Center of Heilongjiang Province, Harbin, China; ^4^ Key Laboratory of Land Surface Pattern and Simulation, Institute of Geographic Sciences and Natural Resources Research, Chinese Academy of Sciences (CAS), Beijing, China

**Keywords:** change, net ecosystem productivity (NEP), influencing factors, terrestrial ecosystem, China

## Abstract

Changes in net ecosystem productivity (NEP) in terrestrial ecosystems in response to climate warming and land cover changes have been of great concern. In this study, we applied the normalized difference vegetation index (NDVI), average temperature, and sunshine hours to drive the C-FIX model and to simulate the regional NEP in China from 2000 to 2019. We also analyzed the spatial patterns and the spatiotemporal variation characteristics of the NEP of terrestrial ecosystems and discussed their main influencing factors. The results showed that (1) the annual average NEP of terrestrial ecosystems in China from 2000 to 2019 was 1.08 PgC, exhibiting a highly significant increasing trend with a rate of change of 0.83 PgC/10 y. The terrestrial ecosystems in China remained as carbon sinks from 2000 to 2019, and the carbon sink capacity increased significantly. The NEP of the terrestrial ecosystem increased by 65% during 2015–2019 compared to 2000–2004 (2) There was spatial differences in the NEP distribution of the terrestrial ecosystems in China from 2000–2019. Taking the line along the Daxinganling-Yin Mountains-Helan Mountains-Transverse Range as the boundary, the NEP was significantly higher in the eastern part than in the western part. Among them, the NEP was positive (carbon sink) in northeastern, central, and southern China, and negative (carbon source) in parts of northwestern China and the Tibet Autonomous Region. The spatial variation of NEP in terrestrial ecosystems increased from 2000 to 2009. The areas with a significant increase accounted for 45.85% and were mainly located in the central and southwestern regions. (3) The simulation results revealed that vegetation changes and CO_2_ concentration changes both contributed to the increase in the NEP in China, contributing 85.96% and 36.84%, respectively. The vegetation changes were the main factor causing the increase in the NEP. The main contribution of this study is to further quantify the NEP of terrestrial ecosystems in China and identify the influencing factors that caused these changes.

## 1 Introduction

The Intergovernmental Panel on Climate Change (IPCC) Sixth Assessment Work Report states that by 2019, the concentration of carbon dioxide (CO_2_) in the atmosphere had reached 409.9 ( ± 0.4) ppm, an increase of 125 ppm over the previous 170 years, and its warming effect is causing climate change and frequent climate disasters (IPCC, 2021). The main sources of carbon dioxide in the atmosphere are fossil fuel combustion, cement production, land use changes, biological respiration, and ocean release. The net ecosystem productivity (NEP) is used to express the net storage of carbon in large-scale ecosystems and can indicate the carbon dioxide exchange between a terrestrial ecosystem and the atmospheric system. Carbon sinks mainly include terrestrial ecosystem photosynthesis, ocean absorption, and organic and inorganic carbon deposited in the land and ocean (Gunter et al., 1998; Houghton, 1998). As a carbon sink for CO_2_, terrestrial ecosystems have become an important component of the global carbon cycle and play an important role in global climate change (Aubinet et al., 2018). Therefore, it is of great significance to accurately evaluate the exchange of CO_2_ between terrestrial ecosystems and the atmosphere in studies of the terrestrial ecosystem carbon cycle.

Many studies have been conducted on the NEP effect on terrestrial ecosystems and have mainly focused on the assessment of the NEP effect, the characteristics, and the mechanisms of the changes. Regarding the assessment of the NEP effects on terrestrial ecosystems, many studies have been conducted on the NEP of terrestrial ecosystems globally and in different countries and regions. In such studies, the average annual NEP has mainly been estimated. Some scholars have focused on the average annual NEP of terrestrial ecosystems per unit area. For example, Zhang et al. (2021) estimated the NEP of the terrestrial ecosystem in Central Asia during 2000–2020 using a combination of the Carnegie Ames Stanford Approach (CASA) model and an empirical model, and its value was determined to be -53.85–108.49 gC/m^2^/y; Lu and Zhuang (2010) estimated the NEP in the midwestern United States from 1948 to 2005 using the terrestrial ecosystem model (TEM), and the result was 87 gC/m^2^/y. Some scholars have focused on the total annual NEP of terrestrial ecosystems. For example, Chinese scholars used the CEVSA, GOSAT, IBIS, CEVSA2, BEPS, TEC, and other models to simulate the average annual NEP of the terrestrial ecosystems in China on different time scales. From 1960 to 2010, the average annual total NEP in different study periods ranged from 0.07 PgC/y to 1.89 PgC/y (Cao et al., 2003; Zhang et al., 2014; Wang et al., 2015; Yang et al., 2016; Yao et al., 2018; He et al., 2019; Zhang et al., 2020; Wang et al., 2021). Nayak et al. (2015) used the CASA model to simulate the NEP of the Indian terrestrial ecosystem from 1981 to 2006 and concluded that the average annual NEP was 10 TgC. Scholars have also paid attention to changes in the NEP of terrestrial ecosystems in different regions and have concluded that the change characteristics of the NEP of terrestrial ecosystems in different regions are different. Zhang et al. (2021) concluded that the NEP in Central Asia decreased at a rate of 6.1 gC/m^2^/10y during 2000–2020. Holland and Brown (1999) concluded that the NEP of the terrestrial ecosystems in the United States and Canada increased from 1987 to 1988; From 1981 to 2006, carbon sources became carbon sinks in India (Nayak et al., 2015). The carbon sink capacity of China’s ecosystems increased from 2000 to 2015 (Zhang et al., 2020). He et al. (2019) revealed the decadal-scale changes in the NEP, that is a decrease of -5.95 TgC/yr^2^ (decreasing sink) during 1982–2000 and an increase of 14.22 TgC/yr^2^ (increasing sink) during 2000–2010. In addition, the influencing mechanism of the NEP changes in terrestrial ecosystems has also attracted the attention of some scholars. Zhao et al. (2020) found that climate change had a positive effect on the increase in the vegetation carbon storage of the global forest ecosystem from 2006 to 2010. However, many scholars have suggested that climate change has had a negative impact on the carbon sink capacity of terrestrial ecosystems (Tian et al., 2011; Mu et al., 2015; Sitch et al., 2015; Biederman et al., 2017; Verduzco et al., 2018). Precipitation and temperature changes may have profound effects on the carbon cycle (Wang et al., 2021).

In summary, even though scholars have conducted a great deal of research on the NEP of terrestrial ecosystems in different regions, the regions involved are still limited and the conclusions are different. Even within the same region, taking China as an example, when the time scale is different, the results are different. Therefore, research on the NEP of terrestrial ecosystems requires further discussion. Regarding the study of the changing trend of the NEP of terrestrial ecosystems, the conclusions also have large differences and need to be discussed. Regarding the impact mechanisms of the changes in the NEP of terrestrial ecosystems, existing studies have mostly focused on the impacts of climate change. However, changes in vegetation and the concentration of CO_2_ are also important factors driving the changes in the NEP, but limited research has been conducted on this relationship. China is located in the middle and high-latitude regions of the Northern Hemisphere, covers a vast area, spans multiple climatic zones, and is characterized by complex and diverse ecological and environmental conditions (Tao et al., 2007). It is a key region for studying the global terrestrial carbon cycle (Liu et al., 2013a).

The main objectives of this study were 1) to use daily meteorological data and NDVI data to drive the C-FIX model to simulate the spatial and temporal variations in the NEP of the terrestrial ecosystems in China from 2000 to 2019, 2) to reveal the factors influencing the NEP changes in the terrestrial ecosystems in China by setting up control experiments, and 3) to provide a scientific basis for revealing the changes in the NEP and their influencing factors in the terrestrial ecosystems in China. Our research also provides evidence for the study of global greenhouse gas concentrations and global climate change.

## 2 Materials and methods

### 2.1 Data sources and processing

The parameters driving the C-FIX model mainly include the NDVI, daily average temperature, daily average radiation, and atmospheric CO_2_ concentration. The data acquisition process was as follows.

#### 2.1.1 Normalized difference vegetation index

The NDVI data used in this study was obtained from the MOD13A3 product, which has a spatial resolution of 1 km×1 km, one image per month, and a total of 12 images throughout the year, and every 35 consecutive images can cover China (row numbers: h23–h29, column numbers: v03–v08). The NDVI data used in this study were from January 2000 to December 2019, with a total of 8400 images. The data were downloaded from the official National Aeronautics and Space Administration (NASA) website (https://ladsweb.nascom.nasa.gov/data/search.html). A total of 240 monthly NDVI products from 2000 to 2019 were finally processed using the ERDAS9.1 software to conduct the format conversion, image merging, and projection conversion. MODIS data for January 2000 was unavailable, so the NDVI data for January 2001 was replaced.

#### 2.1.2 Daily average temperature data

**Figure 1:**
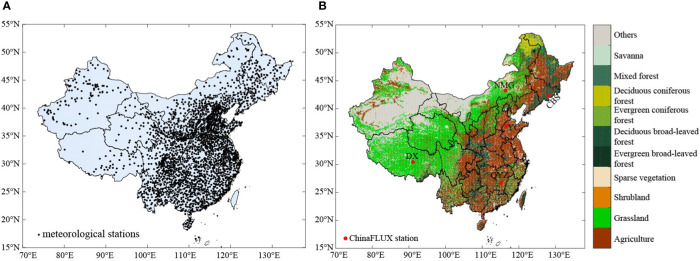
Spatial distribution of meteorological stations **(A)**, vegetation types and spatial distribution of ChinaFLUX station **(B)** in China.

The daily average temperature data used in our analysis were derived from the daily dataset (V3.0) of Chinese national ground meteorological stations, which contains daily observations of basic meteorological elements at 2474 stations in China (**Figure 1A**), including basic, reference, and general meteorological stations, since January 1951. The dataset was produced under strict quality control, the concerns and errors found during the detection were generally verified and corrected manually, and similar “ missing data” phenomena caused by digital omissions were also corrected. In this study, the daily average temperature data in this dataset were selected for use, and the time scale was from 2000 to 2019. Considering the completeness of the data and the accuracy of the results, after eliminating the stations with poor continuity, the average temperature data from 2257 meteorological stations were finally selected for use in the calculations. Spatial interpolation was performed using ArcGIS and the gradient inverse distance square method (GIDS) (Lin et al., 2002), and the spatial resolution was kept consistent with the NDVI data.

#### 2.1.3 Incoming daily global radiation

Since there were relatively few meteorological stations with ground radiation observations in China, this study estimated the incoming daily radiation by the daily sunshine hours. The daily sunshine hours data used were derived from the daily dataset (V3.0) of Chinese national ground meteorological stations.


(1) $$S_{g,d}=(a_{s}+b_{s}\frac{n}{N})R_{a}$$

*S*_*g*,*d*_ : Incoming daily global radiation (MJ/m^2^/day); *n* : Actual sunshine hours (h); *N* : Maximum possible sunshine hours; *R*_*a*_ : Clear sky solar radiation (MJ/m^2^/day); *a*_*s*_=0.25 ; *b*_*s*_=0.50 (Jones, 1992).

The daily *R_a_* can be estimated from the solar constant, the magnetic declination of the Sun, and the position of the day in the year.


(2) $$R_{a}=\frac{24(60)}{\pi }G_{sc}d_{r}[\omega_{s}\sin(\phi )\sin(\delta )+\cos(\phi )\cos(\delta )\sin(\omega_{s})]$$

(3) $$d_{r}=1+0.033\cos(\frac{2\pi }{365}J)$$

(4) $$\delta =0.408\sin(\frac{2\pi }{365}J-1.39)$$

(5) $$\omega_{s}=\arccos[-\tan(\phi )\tan(\delta )]$$

(6) $$N=\frac{24}{\pi }\omega_{s}$$

where: *G*_*sc*_ : Solar constant (0.082); *d*_*r*_ : Relative distance between sun and earth; *ω*_*s*_ : Sunrise and sunset angle (rad); *ϕ* : latitude (rad); *δ* : Solar magnetic declination angle (rad); J: Day order [1-365/366] January 1 takes the day order of 1 (Yan et al., 2016). The calculated *S_g,d_* was spatially interpolated using ArcGIS software with the inverse distance square method (IDS) (Lin et al., 2002), and the spatial resolution was kept consistent with the NDVI data.

#### 2.1.4 Atmospheric CO_2_ concentration data

CO_2_ is a fundamental substance for photosynthesis in vegetation, and an increase in the atmospheric CO_2_ concentration will inevitably affect the photosynthetic outcome of vegetation. Moreover, atmospheric CO_2_ concentration data are one of the parameters driving the C-FIX model. In this study, the global monthly atmospheric CO_2_ mixture concentrations measured during 2000–2019 and reported in the Copenhagen conference proceedings were selected and downloaded from http://co2now.org/.

#### 2.1.5 Land use/cover data

The global land cover data from the Climate Change Initiative-Land Cover (CCI-LC) dataset developed by European Space Agency (ESA), which has a high precision and long time series, were adopted. The data format is TIFF, the coordinate system is WGS1984, and the spatial resolution is 300 m. The land use/cover data for 2000, 2005, 2010, and 2015 were downloaded from the official website of the ESA (http://maps.elie.ucl.ac.be/CCI/viewer/index.php). The data for China’s regional land use/cover in 2000, 2005, 2010, and 2015 were obtained by cutting them with the Chinese area vectorization layer. According to the land use/cover classification system developed by the Food and Agriculture Organization of the United Nations, this product divides the land use types into 22 categories and 36 subcategories. To maintain better data consistency, based on the United States Geological Survey’s (USGS) classification system, the land cover types of the entire country were reclassified into 11 types: evergreen coniferous forest, evergreen broad-leaved forest, deciduous coniferous forest, deciduous broad-leaved forest, mixed forest, shrubland, grassland, agriculture, savanna, sparse vegetation and other (**Figure 1B**).

### 2.2 C-FIX model introduction

The C-FIX model is a light energy utilization model based on Monteith’s theory (Veroustraete, 1994), which enables the simulation of three fundamental carbon cycle components (the gross primary productivity (GPP), net primary productivity (NPP), and NEP) estimated at regional and global scales. In recent years, many scholars have used the C-FIX model to simulate the GPP, NPP, and NEP, and have achieved good simulation results (Lu et al., 2005; Zhang et al., 2011; Yan et al., 2016). For each pixel, daily GPP, NPP, and NEP were estimated according to Eq. (7) to Eq. (14). **Table 1** and **Table 2** list the parameters of the C-Fix model (Veroustraete et al., 2002).

**Table 1:** Description of parameters in the C-FIX model.

Parameter	Significance	Value	Unit
*p(T_atm_)*	Normalized temperature dependency factor [0,1] (Wang, 1996)		[-]
*CO_2_fert*	Normalized CO_2_ fertilization factor (Veroustraete, 1994)		[-]
*ϵ*	Radiation Use Efficiency (RUE)		gC/MJ
*C*	Climatic efficiency (McCree, 1972)	0.48	[-]
*S_g,d_*	Incoming daily global radiation		MJ/m^2^/d
*A_d_*	Autotrophic respiratory fraction (Goward and Dye, 1987)		[-]
*R_h,d_*	Heterotrophic respiration (Maisongrande et al., 1995)		gC/m^2^/d
*fAPAR*	Fraction of absorbed Photosynthetically Active Radiation (PAR) (Asrar et al., 1984)		

ϵ of (8) was assigned by different land use types in **Table 3**. [-] Indicates that there is no unit.

**Table 2:** List of the parameters used in the Eq. (10) to Eq. (14) (Veroustraete et al., 2002).

Parameter	Significance	Value	Unit
*C_1_*	Constant	21.77	
*△H_a,p_*	Activation energy	52750	J/mol
*ΔS*	Entropy of the denaturation equilibrium of CO_2_	704.98	J/K/mol
*R_g_*	Gas constant	8.31	J/K/mol
*T*	Air temperature		K
*T_a_*	Air temperature		°C
*△H_d,p_*	Deactivation energy	211000	J/mol
*τ*	CO_2_/O_2_ specificity ratio		
[*CO_2_*]	CO_2_ concentration in the mesophyll tissue of leaves		ppmv
[*O_2_*]	O_2_ concentration in the mesophyll tissue of leaves	20.9	ppmv
[*CO_2_*]*^ref^*	CO_2_ concentration in the atmosphere	285	ppmv
*K_m_*	Affinity constant for CO_2_ of Rubisco		[%CO_2_]
*K_0_*	Inhibition constant for O_2_		[%O_2_]
*NDVI*	Normalized difference vegetation index		
*k_s,y_*	Heterotrophic respiratory rate coefficient		gC/m^2^/d
*Q_10_*	The relative increase of the respiratory flux	1.5	
	for a 10K increase in temperature *T_a_*		

**Table 3:** Radiation Use Efficiency (ϵ) of vegetation in different land use types (Zhang et al., 2018).

Land use types	Ɛ(gC/MJ)	Land use types	Ɛ(gC/MJ)
Evergreen coniferous forest	1.01	Shrubland	0.83
Evergreen broad-leaved forest	1.26	Grassland	0.61
Deciduous coniferous forest	1.10	Agriculture	0.60
Deciduous broad-leaved forest	1.04	Savanna	0.77
Mixed forest	1.12	Sparse vegetation	0.39
		Others	0.39


(7) $$GPP_{d}=p(T_{atm})\times CO_{2}fert\times \epsilon \times fAPAR\times c\times S_{g,d}$$

(8) $$NPP_{d}=GPP_{d}\times (1-A_{d})$$

(9) $$NEP_{d}=NPP_{d}-R_{h,d}$$

(10) $$p(T_{atm})=\frac{e^{\left(C_{1}-\frac{\Delta H_{a,p}}{R_{g}\cdot T}\right)}}{1+e^{\left(\frac{\Delta S\cdot T-\Delta H_{d,p}}{R_{g}\cdot T}\right)}}$$

(11) $$CO_{2}fert=\frac{\left[CO_{2}\right]-\frac{\left[O_{2}\right]}{2\tau }}{{\left[CO_{2}\right]}^{ref}-\frac{\left[O_{2}\right]}{2\tau }}\frac{K_{m}\left(1+\frac{\left[O_{2}\right]}{K_{0}}\right)+{\left[CO_{2}\right]}^{ref}}{K_{m}\left(1+\frac{\left[O_{2}\right]}{K_{0}}\right)+\left[CO_{2}\right]}$$

(12) fAPAR=1.1638×NDVI−0.1426

(13) $$A_{d}=(7.825+1.145T_{a})/100$$

(14) $$R_{h,d}=k_{s,y}.Q_{10}^{T_{a}/10}$$

The affinity coefficients *K_m_* and *K_0 _* show a temperature dependence according to an Arrhenius relationship:


(15) $$K_{m}=Ae^{\left(-E_{a}/R_{g}T\right)}$$

if: *T_a_* ≥ 15°C then *E_a1_* = 59.4 kJ/mol and *A_1_* = 2.419×10^13^

or if *T_a_<* 15 °C then *E_a2_* = 109.6 kJ/mol and *A_2_* = 1.976×10^22^

The inhibition constant *K_0_* for O_2_ is calculated according to Eq. (16), where *A_0_* = 8240 and *E_a0_* = 13.9135 kJ/mol.


(16) $$\tau =A_{\tau }e^{\left(-E_{a\tau }/R_{g}T\right)}$$

Wherein, *A_τ_* = 7.87×10^-5^ and *E*_*aτ*_ = -42.8969 kJ/mol.


(17) $$k_{s,y}=\frac{\sum_{d=1}^{365}\frac{GPP_{d}}{b_{y}}}{\sum_{d=1}^{365}p{\left(T_{atm}\right)}_{d}}$$

The parameter *b_y_* is the mean annual calibration coefficient of soil heterotrophic respiration, and the value is 1.0.

The specific simulation process was as follows. The 300 m land use/cover data layers for 2000, 2005, 2010, and 2015 were used to present the annual land use/cover data layers for 2000–2004, 2005–2009, 2010–2014, and 2015–2019, respectively. According to the land use/cover data, the radiation use efficiency (ϵ) layer for 2000–2019. For each pixel, the daily average temperature, daily sunshine hours, ϵ, and the concentration of CO_2_ and NDVI were adopted to drive the C-FIX model, and the daily raster layer of the GPP, NPP, and NEP was obtained. The monthly and yearly average values were subsequently calculated *via* numerical integration of the flux functions over the number of days in the considered assessment period, mostly 1 year. When NEP>0, the terrestrial ecosystem is a carbon sink and vice versa.

### 2.3 Controlled experiment method

To reveal the causes of the carbon source/sink effects in China’s terrestrial ecosystems, we analyzed the effects of vegetation changes, climate changes (temperature and radiation), and atmospheric CO_2_ changes on the NEP of China’s terrestrial ecosystems and their contribution rates. In this study, the controlled experiment method was applied, that is, the different driving factors were controlled to simulate the difference between the NEP under the actual scenario and under the controlled terrestrial ecosystems, which was defined as the effect of the controlling factor on the carbon source/sink (**Table 4**).

**Table 4:** Controlled experiments.

Controlled experiments	Input parameters for the Simulated value	Input parameters for the Actual value
Experiment 1 (controlled *NDVI*)	*T_a_*, *S_g,d_*, *[CO_2_]* (2015-2019); *NDVI* (2000-2004)	*T_a_*, *S_g,d_*, *[CO_2_]*, *NDVI* (2015-2019)
Experiment 2 (controlled *T_a_, S_g,d_*)	*[CO_2_]*, *NDVI* (2015-2019); *T_a,_ S_g,d_* (2000-2004)	*T_a_*, *S_g,d_*, *[CO_2_]*, *NDVI* (2015-2019)
Experiment 3 (controlled *[CO_2_]*)	*NDVI*, *T_a_*, *S_g,d_* (2015-2019); *[CO_2_]* (2000-2004)	*T_a_*, *S_g,d_*, *[CO_2_]*, *NDVI* (2015-2019)

Taking the analysis of the impact of the vegetation changes on the NEP as an example, controlled experiment 1 is described in **Table 4**. The temperature, radiation, and atmospheric CO_2_ concentration remained unchanged, and only the NDVI was changed. To avoid the effect of anomalous years, we used the 5-year average values. The average temperature, radiation, and CO_2_ concentration from 2015–2019 and the average NDVI from 2000–2004 were used to drive the C-FIX model to simulate the NEP of the vegetation, which was considered to be the simulated value of the NEP under the controlled experiment. The average temperature, radiation, CO_2_ concentration, and average NDVI during 2015–2019 were used to drive the C-FIX model to simulate the NEP, which was considered to be the actual value. The simulated value was subtracted from the actual value, and the difference was the NEP caused by the vegetation change.


(18) $$\Delta_{variation}=NEP_{actual}{\text{-NEP}}_{\text{simulated}}$$

Where: *Δ_variation_* is the NEP change caused by the vegetation change, *NEP_simulated_* is the simulated NEP value, and *NEP_actual_* is the actual NEP value.

### 2.4 Trend analysis method

A univariate linear regression equation of the GPP, NPP, and NEP (y) and the corresponding time (x) was established:


(19) y=ax+b  (i=1,2,…,n)

where *a* is the linear regression coefficient indicating the rate of change in the GPP, NPP, and NEP. A positive or negative value of *a* indicates that the GPP, NPP, and NEP are increasing or decreasing over time, respectively.

The kappa coefficient is generally used to determine the degree of agreement or accuracy between two images, and its calculation formula is (Cohen, 1960):


(20) $$k=\frac{p_{0}-p_{\text{e}}}{1-p_{e}}$$

Among them, *P_0_* is the sum of the number of samples correctly classified in each category divided by the total number of samples, which is the overall classification accuracy. Assume that the numbers of real samples in each category are *a_1_*, *a_2_*,…, *a_c_*, and that the predicted numbers of samples in each category are *b_1_, b_2_*,…*, b_c_*, and that the total number of samples is *n.* Then:


(21) $$P_{e}=\frac{a_{1}\times b_{1}+a_{2}\times b_{2}+\ldots +a_{c}\times b_{c}}{n\times n}$$

The kappa coefficient calculation results are -1–1, but usually, the kappa falls between 0 and 1, which can be divided into five groups to indicate the different levels of consistency: 0.0–0.20, very low consistency; 0.21–0.40, general consistency; 0.41–0.60, moderate consistency; 0.61–0.80, high consistency; and 0.81–1 almost identical (Stehman, 1996).

### 2.5 Statistical analysis

The error analysis of simulated data was conducted by comparison with the measurements. The mean error, root mean square error (*RMSE*), and correlation coefficient (*R*) was used, and calculated as follows:


(22) $$RMSE=\sqrt{\frac{1}{\text{n}}\sum_{i=1}^{n}{\left(x_{i}-y_{i}\right)}^{2}}$$

where *x_i_* represents the simulated data; *y_i_* indicates the actual data; *x* is the average value of the interpolated data; *y* is the average value of the measured data, and *n* represents the number of the sample.

## 3 Results and analysis

### 3.1 Verification of C-FIX model simulation results

In order to verify the simulation accuracy of the C-FIX model more precisely, the GPP, NPP, and NEP were verified.

**Figure 2:**
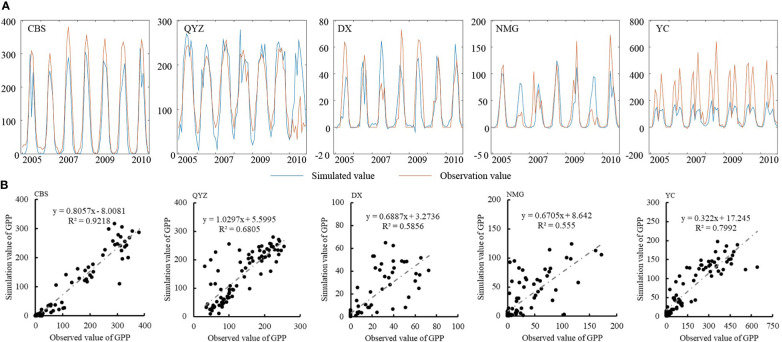
Verification of the GPP (unit: gC/month) **(A)** trend comparison **(B)** the scatter plot of simulated GPP and observations.

Verification of GPP: The monthly measured GPP data were obtained from eight stations in the China Terrestrial Ecosystem Flux Observation and Research Network (ChinaFLUX), among which four were forest stations (CBS, QYZ, DHS, and XSBN), three were grassland stations (DX, NMG, and HB), and one was a cultivated land station (YC). When selecting the verification points, the locations of the verification points were taken into account, that is, they covered the four directions of China, and the vegetation types at the verification points were considered, including forest, grassland, and agriculture. Finally, the monthly measured GPP data for stations CBS, QYZ, DX, YC, and NMG from 2004 to 2010 were selected for verification. According to the latitude and longitude of each observation station, the monthly simulated GPP values of the grid from 2004 to 2010 were extracted, and the accuracy of the simulated values was verified using the trend comparison (**Figure 2A**) and the scatter plot (**Figure 2B**). The R^2^ between the simulated values and measured values at the five stations are 0.92, 0.68, 0.59, 0.56, and 0.78, and they all passed the 0.01 probability level test, indicating that the trends of the simulated and measured values are basically the same. The RMSE of the five stations is 31.71 gC/m^2^/m, 9.71 gC/m^2^/m, 1.65 gC/m^2^/m, 0.59 gC/m^2^/m, and 102.55 gC/m^2^/m, indicating that stations CBS, QYZ, DX, and NMG are within 10% of the measured value, and station YC is greater than 10%. Thus, the simulations for stations CBS, QYZ, DX, and NMG have a higher accuracy, and the simulation for station YC has a slightly poorer accuracy. In summary, the C-FIX model can simulate the terrestrial ecosystem GPP well. Specifically, the vegetation ecosystem GPP simulation accuracy is higher for the forest and grassland (**Figure 2**).

Verification of NPP: The research results of the annual mean NPP of the terrestrial ecosystems in China in recent years are further summarized in **Table 5**. It can be seen that the models used include the BEPS, C-FIX, GEOPRO, M-SDGVM, CASA, MuSyQ-NPP, LPJ-DGVM, and the annual average NPP is between 1.92 and 4.37 PgC/y (**Table 5**). A numerical comparison could not be conducted due to the different time scales of the simulations. However, the simulated values of the BEPS, GEOPRO, MuSyQ-NPP, and LPJ-DGVM are relatively low, while those of the C-FIX and M-SDGVM are slightly higher. The CASA simulation results show that the average annual NPP of the regional terrestrial ecosystem in China was 3.38–4.35 PgC/y from 1981 to 2008, 2.25–2.62 PgC/y from 2001 to 2010, and 2.93 PgC/y in 2015, indicating inter-annual fluctuations. Based on the simulation results of the C-FIX model, the average annual NPP of the regional terrestrial ecosystem in China from 2000 to 2019 was 3.34 PgC/y. Compared with previous studies, our simulation results are less than the maximum value and greater than the minimum value, i.e., within a reasonable range. The simulation results presented in this study are credible.

**Table 5:** Summary of studies on NPP in China.

Methods	Study periods	NPP ranges (PgC/y)	References
BEPS model	2001	2.24	Feng et al. (2007)
C-FIX model	2003	4.37	Chen et al. (2007)
GEOPRO model	2000-2004	2.42-2.84	Gao and Liu (2008)
M-SDGVM	1981-2000	3.30	Mao et al. (2010)
CASA model	1981-2008	3.38-4.35	Chen et al. (2011)
BEPS model	2000-2010	2.63-2.84	Liu et al. (2013b)
CASA model	2001-2010	2.25-2.62	Pei et al. (2013)
CASA model	2015	2.928	Li et al. (2018)
MuSyQ-NPP	1982-2012	1.92-2.73	Wang et al. (2019)
LPJ-DGVM model	1961-2016	2.8-3.1	Huang et al. (2019)
C-FIX model	2000-2019	3.34	This study

**Figure 3:**
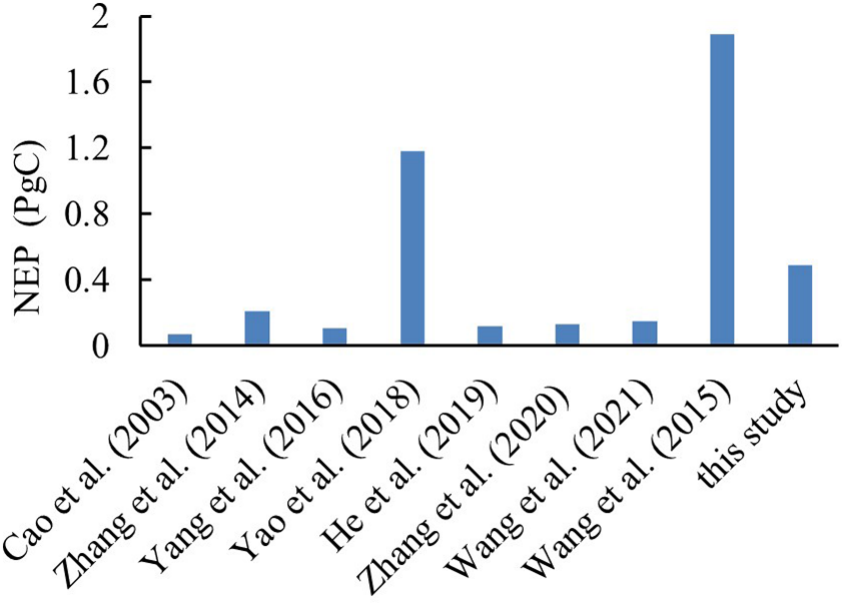
Verification of the NEP.

Verification of NEP: **Figure 3** presents a comparison of our C-FIX model simulation results for the terrestrial ecosystem NEP in China and the results of previous studies. The results of different studies differ greatly. The simulation results of the CEVSA2, BEPS, TEC, IBIS, GOSAT, and other models are small, while the NEP values observed using vorticity correlation measurement methods are high. In summary, different researchers have applied different time scales, different model parameters, and different driving data sources, resulting in different results. The results of the previous studies show that the NEP in the terrestrial ecosystems in China was 0.07–1.89 PgC; however, in this study, the NEP values of the terrestrial ecosystems in China during 2000–2019 were calculated to be 0.6–1.38 PgC. The calculated results are within the NEP thresholds of previous studies, further demonstrating the accuracy of our results.

### 3.2 Temporal and spatial variation characteristics of carbon sources/sinks in China’s terrestrial ecosystems from 2000 to 2019

#### 3.2.1 Temporal patterns and change characteristics of the GPP, NPP, and NEP

**Figure 4:**
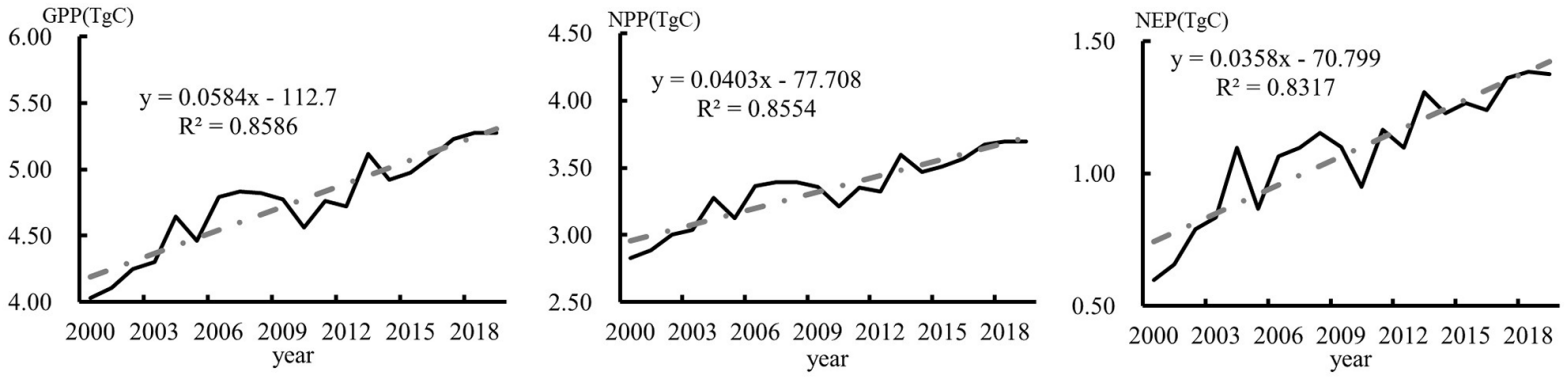
Changes in the GPP, NPP, and NEP in China from 2000 to 2019.

The mean GPP, NPP, and NEP values of the regional terrestrial ecosystems in China from 2000 to 2019 were 4.75 PgC, 3.34 PgC, and 1.08 PgC, respectively. **Figure 4** shows the temporal variations in the GPP, NPP, and NEP in China’s regional terrestrial ecosystems during 2000–2019. It shows that all three exhibited increasing trends with annual coefficients of variation of 7.86%, 7.72%, and 24.45%, respectively, indicating that the interannual variations in the GPP and NPP in China’s regional terrestrial ecosystems were small. The rates of increase of the GPP, NPP, and NEP of the regional terrestrial ecosystems in China from 2000 to 2019 were 0.86 PgC/10 y, 0.86 PgC/10 y, and 0.83 PgC/10 y, respectively (**Figure 4**), and the equations passed the 0.01 probability level test (R^2^ critical value of 0.608). Furthermore, **Figure 4** shows that the terrestrial ecosystems in China remained a carbon sink during 2000–2019, and their carbon sink capacity increased significantly. Analysis of the variance of the annual mean NEP of the terrestrial ecosystems in China during 2015–2019 and 2000–2005 revealed that there was a significant difference between these two periods at the 0.01 level, further confirming that the NEP of China’s regional terrestrial ecosystems changed significantly during 2000–2019.

#### 3.2.2 Spatial patterns and change characteristics of the GPP, NPP, and NEP

**Figure 5:**
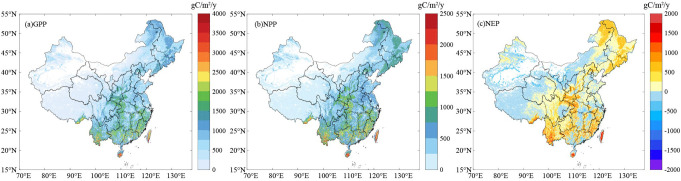
Spatial distributions of the **(A)** GPP, **(B)** NPP, and **(C)** NEP in China from 2000 to 2019.

**Figure 5** shows the spatial patterns of the GPP, NPP, and NEP of the terrestrial ecosystems in China from 2000 to 2019. **Figure 5A** showed that the GPP, NPP, and NEP of the terrestrial ecosystems in China during 2000–2019. Taking the line along the Daxinganling-Yin Mountain-Helan Mountain-Hengduan as the boundary, the values were significantly higher in the eastern part than in the western part. Most of the high-value annual average GPP areas had values of >1750 gC/m^2^, while the low-value areas in the west had values of<500 gC/m^2^, and the values in the central and northeastern regions were basically within 750–1500 gC/m^2^. Most of the high-value annual average NPP areas had values of >1500 gC/m^2^, while the low-value areas in the west had values of<250 gC/m^2^, and the values in the central and northeastern regions were basically within 500–1250 gC/m^2^ (**Figure 5B**). Most of the high-value annual average NEP areas in the southeastern region had values of >1000 gC/m^2^, while the low-value areas in the west had values of<-500 gC/m^2^, and the values in the central and northeastern regions were basically within 0–750 gC/m^2^ (**Figure 5C**).

**Figure 6:**
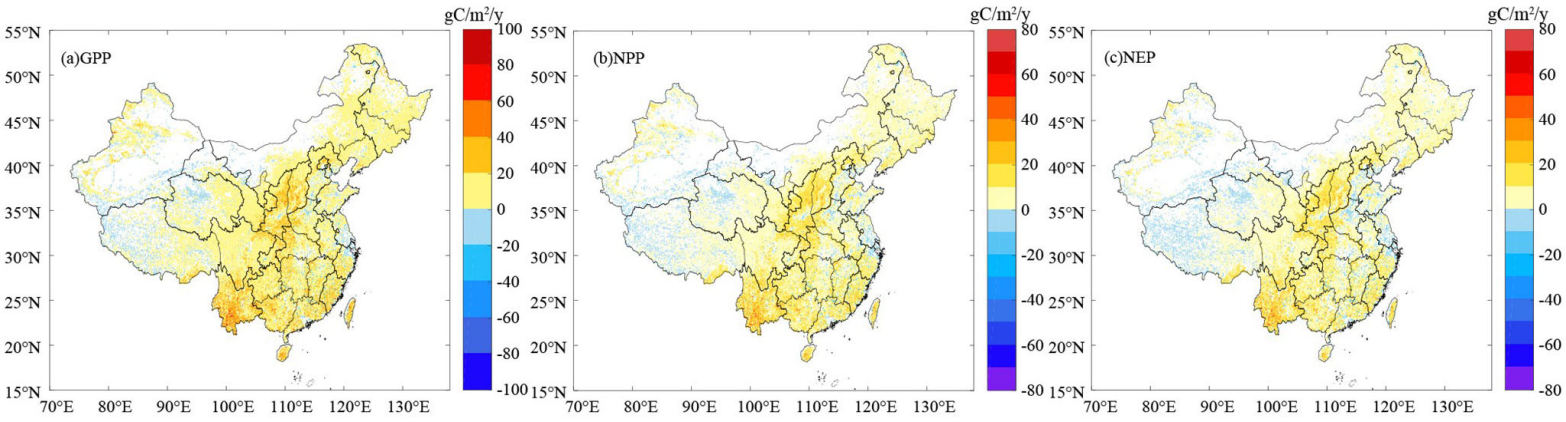
Spatial variations in the **(A)** GPP, **(B)** NPP, and **(C)** NEP in China from 2000 to 2019.

**Figure 6** shows the spatial variation characteristics of the annual average GPP, NPP, and NEP of the terrestrial ecosystems in China from 2000 to 2019. The spatial changes in the GPP, NPP, and NEP were consistent, and their rates of change were high in the south and east and low in the north and west. Their rates of change were all dominated by significantly increasing trends (**Figures 6A–C**). The areas with significant increases in the GPP, NPP, and NEP of the terrestrial ecosystems accounted for 50.51%, 50.67%, and 45.85% of the total area of the country, respectively, and the areas with high values of significant increase were mainly located in the central and southwestern regions. The areas with significant decreases in the GPP, NPP, and NEP of the terrestrial ecosystems accounted for 18.23%, 18.51%, and 36.01% of the total area of the country, respectively, and the areas with significant decreases were mainly sporadically distributed in eastern and northwestern China. The carbon sink capacity in central and southwestern China continuously increased, while in eastern China and some parts of northwestern China, the carbon sink capacity decreased even though these areas were carbon sink areas.

### 3.3 Analysis of influencing factors of NEP changes in terrestrial ecosystems in China from 2000 to 2019

Three sets of controlled experiments were conducted to investigate the factors influencing the NEP changes in the terrestrial ecosystems in China. The results showed that vegetation changes and CO_2_ concentration changes caused the increase in the NEP of the terrestrial ecosystems in China from 2000 to 2019. However, climate change caused the decrease in the NEP. Vegetation changes caused the largest increase in the NEP in the terrestrial ecosystems in China (0.49 PgC), which was 2.3 times the increase in the NEP caused by changes in the CO_2_ concentration (**Table 6**). The effect of climate change on the NEP was 26.53% and 61.90% that of the effects of the changes in the vegetation and CO_2_ concentration on the NEP. Assuming that the absolute value of the NEP caused by each factor was 100%, the change in the NEP caused by vegetation changes accounted for 85.96%, while those caused by climate change and changes in the atmospheric CO_2_ concentration accounted for -22.80% and 36.84%, respectively, implying that the vegetation changes were the main cause of the increase in the carbon absorption in the terrestrial ecosystems in China.

**Table 6:** Results of controlled experiments (TgC=10^12^ gC).

Controlled experiment	Simulated value	Actual value	Δ_variation_
Experiment 1 (controlled *NDVI*)	0.83	1.32	0.49
Experiment 2 (controlled *T_a_, S_g,d_*)	1.45	1.32	-0.13
Experiment 3 v(controlled *[CO_2_]*)	1.11	1.32	0.21

^Δ^The NEP change caused by the vegetation/climate/atmospheric CO2 change.

**Figure 7:**
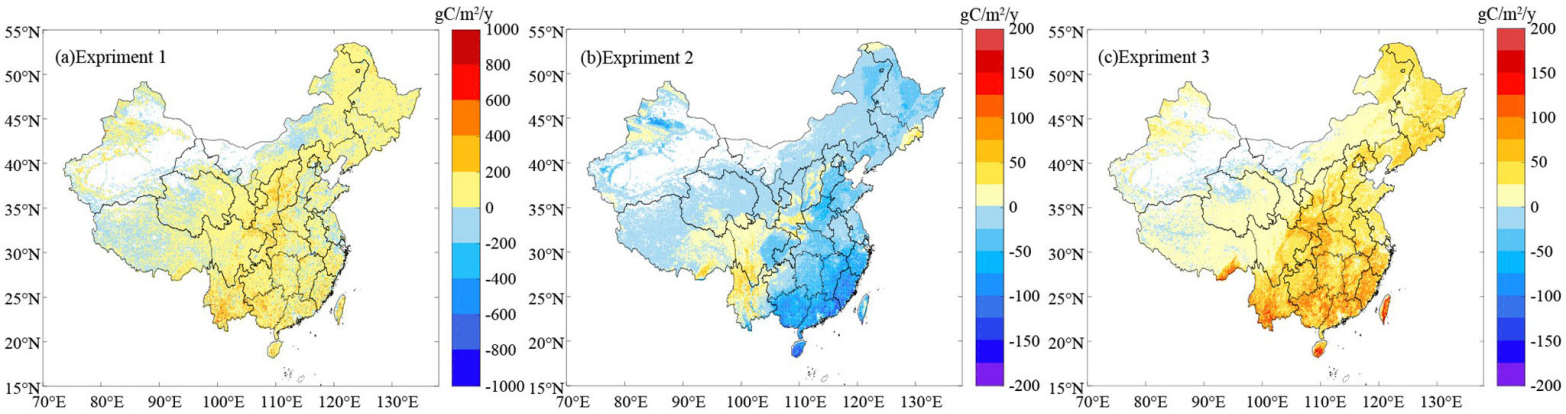
Spatial distribution of the NEP changes based on the controlled trials: **(A)** vegetation changes; **(B)** climate change; and **(C)** CO_2_ density changes.

The spatial patterns of the controlled experiment results show that the vegetation changes increased the NEP of the terrestrial ecosystems in 61.53% of the entire country, and the high-value areas were located in the Daxinganling-Taihang Mountains-Qinling area and southern China. The vegetation changes only caused a decrease in the NEP of the terrestrial ecosystems in 16.96% of the total area, mainly in Hulunbeier, Inner Mongolia, and parts of eastern China (**Figure 7A**). Climate change caused an increase in the NEP of the terrestrial ecosystems in 14.89% of the total area, mainly in Yunnan Province in southwestern China, while it caused a decrease in the NEP in 63.60% of the terrestrial ecosystems, mainly in northeastern, central, and eastern China (**Figure 7B**). The changes in the CO_2_ concentration caused an increase in the NEP in 73.82% of the regional terrestrial ecosystems in China, and the high-value areas were located in southeastern China; changes in the CO_2_ concentration caused a decrease in the NEP in only 4.67% of the region, mainly sporadically distributed in northwestern China and the Qinghai-Tibetan Plateau (**Figure 7C**).

Based on the spatial distribution characteristics, the CO_2_ concentration changes, vegetation changes, and climate change caused increases or decreases in the NEP of the terrestrial ecosystems in some regions. Therefore, the factors causing the changes in the NEP of the terrestrial ecosystems in the different administrative regions of China were revealed through the control experiments (**Table 7**). The vegetation changes were the main factor causing the changes in the NEP of the terrestrial ecosystems in northeastern, northern, eastern, central, southern, northwestern, and southwestern China. The proportion of the provinces and cities in which the NEP of the terrestrial ecosystems was affected by vegetation was 93.97%, mainly in Heilongjiang Province, the Inner Mongolia Autonomous Region, Guangxi Province, Shaanxi Province, Yunnan Province, and Sichuan Province. Jiangsu Province and Taiwan Province were affected by the changes in CO_2_ concentration, accounting for 6.06%.

**Table 7:** NEP changes in each administrative region in China under the control of the experimental situation (TgC).

Administrative	NDVI	CO_2_	Climate	Influencing	Administrative	NDVI	CO_2_	Climate	Influencing
district	change	change	change	factors	district	change	change	change	factors
Heilongjiang	27.23	14.49	-9.45	▲	Henan	11.73	6.27	-5.13	▲
Liaoning	8.5	4.67	-2.78	▲	Hunan	20.13	10.88	-4.28	▲
Jilin	10.22	6.15	-1.8	▲	Hubei	18.04	8.76	-4.28	▲
Beijing	1.52	0.61	-0.08	▲	Guangxi	36.3	15.84	-16.47	▲
Tianjin	0.3	0.23	-0.25	▲	Guangdong	18.23	10.59	12.95	▲
Hebei	11.55	5.36	-3.14	▲	Hainan	4.26	2.56	-3.14	▲
Shaanxi	21.83	5.09	-0.54	▲	Hong Kong	-0.02	0.003	-0.01	▲
Inner Mongolia	30.38	14.27	-12.38	▲	Shanxi	30.45	8.85	0.2	▲
Shandong	6.3	3.92	-4.22	▲	Gansu	20.57	4.15	-1.78	▲
Jiangsu	0.15	2.4	-2.04	•	Ningxia	3.72	0.57	-0.55	▲
Anhui	8.37	5.32	-3.64	▲	Qinghai	7.81	1.54	-1.66	▲
Shanghai	-0.2	0.08	-0.13	▲	Xinjiang	12.78	4.18	-6.49	▲
Zhejiang	7.82	4.9	-5.44	▲	Yunnan	62.2	22.59	4.06	▲
Jiangxi	14.8	8.9	-10.25	▲	Sichuan	39.32	13.46	1.03	▲
Taiwan	2.74	2.82	-1.34	•	Guizhou	23.41	9.23	-2.37	▲
Fujian	12.38	8.11	-9.94	▲	Chongqing	11.05	3.93	-1.62	▲
					Tibet	13.42	6.05	0.35	▲

No data for Macao; ▲: NDVI; ■, Climate; •, CO_2_.

## 4 Discussions

**Figure 8:**
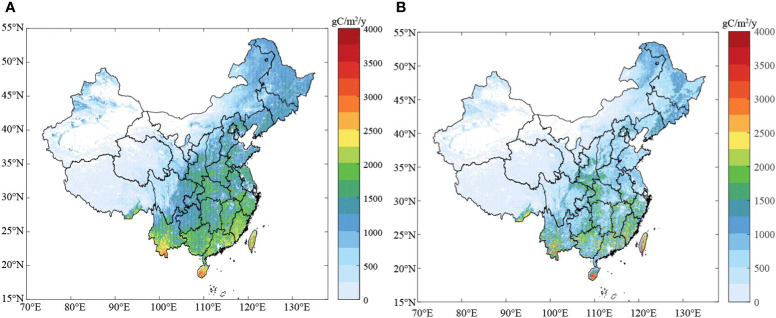
Spatial distribution of the NEP under different radiation use efficiencies: **(A)** ϵ=1.1; **(B)** ϵ (**Table 2**).

(1) In this study, the spatial and temporal patterns and change characteristics of the NEP of the terrestrial ecosystems in China during 2000–2019 were analyzed based on simulations of the GPP, NPP, and NEP of the terrestrial ecosystems conducted using the C-FIX model of light energy utilization, and the factors influencing the changes in the NEP of the terrestrial ecosystems in China were further analyzed. The results of this study not only provide and update basic data on carbon stocks in regional terrestrial ecosystems in China but also provide a basis for quantitative analysis of the carbon stock changes in terrestrial ecosystems in China.

(2) Some scholars have studied the ratio of the NPP to the GPP. For example, Zhang et al. (2009) used MODIS data for 2000–2003 to calculate the ratio of the NPP to the GPP of global terrestrial ecosystems and obtained a value of 0.52. Wang (2012) reported that the annual average ratio of the NPP to the GPP for the terrestrial ecosystems in east Asia during 1949–2008 was 0.604 based on the atmospheric-vegetation interaction model version 2 (AVIM2). Scholars have also calculated the ratio of the NPP to the GPP for the forest system. Landsberg et al. (2020) used the forest grow model 3-PG to analyze the ratio of the NPP to the GPP at more than 200 forest stations. Their results revealed that the range of the values was mostly 0.4–0.6. Waring et al. (1998) reported that the ratio of the NPP to the GPP of forest ecosystems was between 0.22 and 0.79. By comparing the NPP/GPP ratios of different regions simulated using the C-FIX model, Song et al. (2021) determined that the NPP/GPP ratio of terrestrial ecosystems in the Gannan region of China in 2019 was 0.77. It can be seen that due to the different data resources and different simulation methods used, the results of the NPP/GPP ratios of different ecosystems are different, with values of basically between 0.22 and 0.79. In this study, the annual average NPP/GPP ratio of China’s terrestrial ecosystems from 2000 to 2019 calculated using the C-FIX model was 0.70. Based on a comprehensive comparison, the NPP/GPP ratio simulated using the C-FIX model is high, but it is within a reasonable range. According to the test results of this study, the GPP simulated using the C-FIX model is slightly lower, while the NPP is slightly higher, which is the reason why the NPP/GPP ratio is high.

(3) The C-FIX model is a light energy utilization model, and its Radiation Use Efficiency (ϵ) is an important parameter for constraining the accuracy of the model (Yuan et al., 2014). Its value is influenced not only by the vegetation type but also by the uniformity of the vegetation cover, and there are differences in the ϵ values of different vegetation types (Yan et al., 2016). Thus far, while most scholars have used the C-FIX model to simulate the GPP, NPP, and NEP of terrestrial ecosystems, the radiation use efficiency (ϵ) of vegetation has been set as a constant, i.e., the differences in the radiation use efficiencies of different types of vegetation were not considered. For example, in previous studies, when simulating the GPP, NPP, and NEP in the ecosystems in China (Chen et al., 2007), western China (Lu et al., 2005), the Gannan region of China (Song et al., 2021), and other places, the ϵ value was set as 1.1 gC/MJ. However, as the accuracy of the simulation has improved in recent years, some scholars noticed the difference in the ϵ values of different types of vegetation. When Yan et al. (2016) used the C-FIX model to simulate the GPP, NPP, and NEP of a terrestrial ecosystem, they divided the vegetation types in the region into six types (i.e., cultivated land, grassland, closed forest, shrub, open forest, and other forests), and the ϵ value was set according to the vegetation type. Based on the vegetation types in China and referring to the results of previous research, in this study, the vegetation types were divided into 11 types with different ϵ values (**Table 3**). In order to compare the two different assignments, the difference between the simulation results of the two scenarios was further assessed. When the radiation use efficiency of all of the vegetation was assigned a constant value (ϵ=1.1), the simulated total annual GPP of the terrestrial ecosystem in China during 2000–2019 was 6.76 PgC. In contrast, when the ϵ was assigned according to the vegetation types, the GPP was 4.75 PgC. It can be seen that there is a difference between the two results. When the ϵ value is assigned as a constant, the simulation result is high, and the difference between the two is 2.01 PgC. The spatial distributions of the simulated total annual GPP of the terrestrial ecosystems in China during 2000–2019 obtained using the two methods are shown in **Figure 8**. Through this comparison, it can be seen that when ϵ is assigned according to the vegetation type, the spatial differentiation of the GPP is significantly improved. Therefore, assigning different radiation use efficiency values to the different vegetation types makes the simulated values more accurate and the spatial differentiation clearer.

(4) Until now, although researchers have simulated the carbon source/sink changes in terrestrial ecosystems in different regions in China and abroad, few studies have analyzed the influencing factors of these changes. Studies of the factors influencing NEP changes have mainly focused on climate change, and the results are different. For example, Cao et al. (2003) analyzed climate change and the changes in the NEP of the terrestrial ecosystem in China from 1981 to 1998 and reported that climate change weakened the terrestrial ecosystem’s carbon sink capacity. He et al. (2019) reported that the carbon sink capacity in China increased from 2000 to 2010, and this shift was mainly influenced by the enhanced summer monsoon, with the climate effect accounting for 56.3%. Compared with previous studies, this study not only focused on the impacts of climate change on the NEP but also revealed the impacts of vegetation changes and CO_2_ concentration changes on the NEP. The results of this study provide a reference for studying the NEP mechanism in regional terrestrial ecosystems.

(5) In this study, by using the controlled experiment method, it was concluded that the increase in the NEP in more than 90% of the provinces in China was influenced by vegetation changes, among which the NEP in Yunnan, the Inner Mongolia Autonomous Region, and Shaanxi and Guangxi provinces was most significantly influenced by the vegetation changes. To further verify these results, we reviewed the white paper *Twenty Years of Returning Farmland to Grassland in China (1999–2019)* and found that 515 million acres of cropland were returned to forest and grassland in China in the past 20 years, of which the increase in forest land was 502 million acres, and data on vegetation changes in the major provinces in China were obtained (**Table 8**). As can be seen from **Table 8**, in Yunnan Province, the Inner Mongolia Autonomous Region, Shaanxi Province, and the Guangxi Zhuang Autonomous Region 107.5 million acres have been afforested since 1999, accounting for 21.41% of the total reforestation area in China. We conclude that the reforestation area in these four provinces accounted for 20% of the total reforestation area in China, and the woodland area increased significantly (**Table 8**). Moreover, the four administrative regions of Yunnan Province, the Inner Mongolia Autonomous Region, Shaanxi Province, and the Guangxi Zhuang Autonomous Region also experienced the largest changes in the terrestrial carbon sink capacity due to vegetation changes, exhibiting good consistency. This also indicates that the research results of this study are consistent with reality.

**Table 8:** Afforestation area in Yunnan Province, Inner Mongolia Autonomous Region, Shaanxi Province, and Guangxi Province (10,000 mu).

Administrative district	Reforestation area	Area of farmland to forest	Reforestation area of barren hills	Forestation of closed hills
Yunnan Province (2000-2015)	1813.93	533.10	1060.33	220.50
Inner Mongolia Autonomous Region (2000-2013)	4261.00	1383.00	2547.00	331.00
Shaanxi Province (2000-2018)	3694.50	1867.50	1932.70	239.50
Guangxi Zhuang Autonomous Region (2001-2018)	1536.70	402.00	975.70	159.00

**Figure 9:**
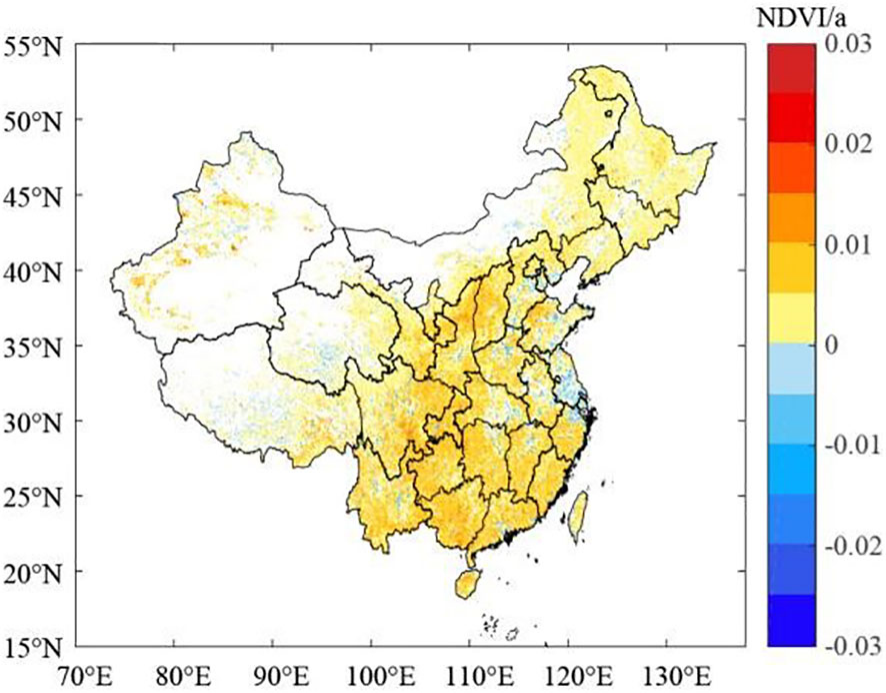
Spatial variations in the NDVI in China from 2000 to 2019.

Based on the NDVI data for China downloaded from the MODIS website, we calculated the rate of change of the NDVI in China from 2000 to 2019 (**Figure 9**). The results show that the NDVI increased in 64.66% of China. This further confirms that the state of the surface vegetation in China improved from 2000 to 2019, and these vegetation changes were the main factor leading to the maintenance of the carbon sinks in the regional terrestrial ecosystems in China and the significant increase in the carbon sink capacity.

(6) In this study, during image data processing, when layer overlay, cropping, and raster calculation were performed, more complicated data processing and calculation processes such as projection conversion, graphic correction, vector data and raster data conversion, boundary alignment, and spatial statistics were required. The mapping process also involved the conversion of the geographic coordinate system, all of which affected the accuracy of the simulation results.

## 5 Conclusions

(1) The mean GPP, NPP, and NEP values of the regional terrestrial ecosystems in China from 2000 to 2019 were 4.75 PgC, 3.34 PgC, and 1.08 PgC, respectively, exhibiting highly significant increasing trends with rates of change of 0.86 PgC/10 y, 0.86 PgC/10 y, and 0.83 PgC/10 y, respectively. The terrestrial ecosystems in China were maintained as carbon sinks during 2000–2019, and their carbon sink capacity increased significantly.
(2) The GPP, NPP, and NEP of China’s terrestrial ecosystems during 2000–2019 exhibited high spatial variability, i.e., high in the south and east and low in the north and low west. Take the Daxinganling-Yin Mountain-Helan Mountain-Hengduan Mountains line as the boundary, the values were significantly higher in the eastern part than in the western part. The spatial change rates of the GPP, NPP, and NEP mainly increased. The areas with significant increases were distributed in central and southwestern China, while the areas with significant decreases were sporadically distributed in eastern and northwestern China.
(3) Vegetation changes and CO_2_ concentration changes caused the increase in the NEP in the terrestrial ecosystems in China during 2000–2019, while climate change had the opposite effect, with contribution rates of 85.96%, 36.84%, and -22.80%, respectively, indicating that vegetation changes were the main reason for the increase in the carbon absorption in the terrestrial ecosystems in China. The vegetation changes increased terrestrial ecosystem NEP in 63.60% of China, and the high-value areas were located in the Daxinganling-Taihang Mountains-Qinling area and southern China.
(4) During 2000–2019, the carbon source/sink capacity of the terrestrial ecosystems in more than 90% of the provinces and cities was affected by vegetation changes, and the vegetation changes led to an increase in the carbon sink capacity in 31 provinces and cities, except for Shanghai and Hong Kong. The changes in the CO_2_ concentration led to an increase in the carbon sink capacity in the entire country, while climate change weakened the carbon sink capacity.

## Data availability statement

The raw data supporting the conclusions of this article will be made available by the authors, without undue reservation.

## Author contributions

YH, analysis of data and drafts the manuscript. FW and LZ approve the final manuscript. JZ, make suggestions for revision. HZ and NW, performed the statistical analysis and visualization. JG and YZ, review of literature and data curation. WZ, revise the manuscript. All authors contributed to the article and approved the submitted version.
