# Corrigendum: Changes and net ecosystem productivity of terrestrial ecosystems and their influencing factors in China from 2000 to 2019

**DOI:** 10.3389/fpls.2023.1259137

**Published:** 2023-08-23

**Authors:** Yutao Huang, Fang Wang, Lijuan Zhang, Junfang Zhao, Hong Zheng, Fan Zhang, Nan Wang, Jiakai Gu, Yufeng Zhao, Wenshuai Zhang

**Affiliations:** ^1^ Heilongjiang Province Key Laboratory of Geographical Environment Monitoring and Spatial Information Service in Cold Regions, Harbin Normal University, Harbin, China; ^2^ State Key Laboratory of Severe Weather, Chinese Academy of Meteorological Sciences, Beijing, China; ^3^ Laboratory of Climate Application, Climate Center of Heilongjiang Province, Harbin, China; ^4^ Key Laboratory of Land Surface Pattern and Simulation, Institute of Geographic Sciences and Natural Resources Research, Chinese Academy of Sciences (CAS), Beijing, China

**Keywords:** change, net ecosystem productivity (NEP), influencing factors, terrestrial ecosystem, China

In the published article, there was an error in **Figure 2** as published. **Figure 2** header has an error. **Figure 2B** is not a histogram, it should be the scatter plot of simulated GPP and observations. The corrected **Figure 2** and its caption “**Figure 2** Verification of the GPP (unit: gC/month) (A) trend comparison (B) the scatter plot of simulated GPP and observations” appear below.

**Figure 2:**
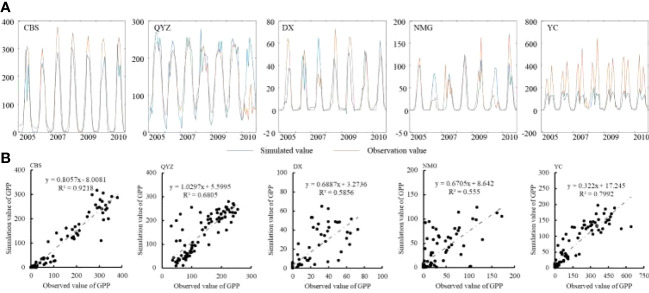
Verification of the GPP (unit: gC/month) **(A)** trend comparison **(B)** the scatter plot of simulated GPP and observations.

In the published article, there was an error. As the title of **Figure 2**, section 3.1 has been modified, the citations of **Figure 2** in the text have also been updated to reflect this

A correction has been made to **3.1 Verification of C-FIX model simulation results**, *Verification of GPP*. This sentence previously stated:

“According to the latitude and longitude of each observation station, the monthly simulated GPP values of the grid from 2004 to 2010 were extracted, and the accuracy of the simulated values was verified using the trend comparison (**Figure 2A**) and histogram (**Figure 2B**).”

The corrected sentence appears below:

“According to the latitude and longitude of each observation station, the monthly simulated GPP values of the grid from 2004 to 2010 were extracted, and the accuracy of the simulated values was verified using the trend comparison (**Figure 2A**) and the scatter plot (**Figure 2B**).”

In the published article, there was an error. In section 3.1, the first sentence of the Verification of NPP has errors. The research results of the annual mean NPP of the terrestrial ecosystems in China in recent years are further summarized in Table 5 instead of Table 4.

A correction has been made to **3.1 Verification of C-FIX model simulation results**, *Verification of NPP*. This sentence previously stated:

“Verification of NPP: The research results of the annual mean NPP of the terrestrial ecosystems in China in recent years are further summarized in Table 4.”

The corrected sentence appears below:

“Verification of NPP: The research results of the annual mean NPP of the terrestrial ecosystems in China in recent years are further summarized in Table 5.”

The authors apologize for these errors and state that these do not change the scientific conclusions of the article in any way. The original article has been updated.

